# Supplementary material for: Algorithm-Based Linearly Graded Compositions of GeSn on GaAs (001) via Molecular Beam Epitaxy
Source: Nanomaterials (Basel). 2024 May 22;14(11):909. doi: 10.3390/nano14110909 (PMC11173740; doi:10.3390/nano14110909)
Supplement: Supplementary file 1 [file nanomaterials-14-00909-s001.zip › nanomaterials-3006831-supplementary.pdf]

Supplementary Material

Calbi Gunder[1*], Mohammad Zamani-Alavijeh[4], Emmanuel Wangila[1], Fernando Maia de Oliveira[2], Aida Sheibani[4], Serhii Kryvyi[2], Paul C. Attwood[3], Yuriy I. Mazur[2], Shui-Qing Yu[2,5], Gregory J. Salamo[2,4]

[1]*Materials Science and Engineering, University of Arkansas, Fayetteville, AR 72701, USA*
[2]*Institute for Nanoscience and Engineering, University of Arkansas, Fayetteville, AR 72701, USA*
[3]*Gunder and Attwood Armories, El Dorado Springs, MO 64744, USA*
[4]*Department of Physics, University of Arkansas, Fayetteville, AR 72701, USA.*
[5]*Department of Electrical Engineering, University of Arkansas, Fayetteville, AR 72701, USA*

1. Obtaining Eq. 4 that provides the Sn composition from the RSM data:

The relaxed lattice parameter a_0^{GeSn} of the GeSn alloy and its set of elastic constants, C_{11}^{GeSn} and C_{12}^{GeSn}, are described in terms of the Vegard's law by:

$$a_0^{GeSn} = xa_0^{Sn} + (1-x)a_0^{Ge} \qquad Eq.(S1)$$

$$C_{11,12}^{GeSn} = xC_{11,12}^{Sn} + (1-x)C_{11,12}^{Ge} \qquad Eq.(S2)$$

The out-of-plane strain, $\varepsilon_\perp^{GeSn}$, can be computed from the in-plane strain, $\varepsilon_{||}^{GeSn}$, by:

$$\varepsilon_\perp^{GeSn} = (-2C_{12}^{GeSn}/C_{11}^{GeSn})\varepsilon_{||}^{GeSn} \qquad Eq.(S3)$$

through the Poisson's ratio. The value reported by Albani et al. for each constant is presented in Table S1 [1].

Table S1: Relaxed lattice parameters $a_0^{Ge,Sn}$ and elastic constants $C_{11,12}^{Ge,Sn}$ of Ge and Sn.

a_0^{Sn}	0.6489 nm
a_0^{Ge}	0.5658 nm
C_{11}^{Sn}	69 GPa
C_{12}^{Sn}	29.3 GPa
C_{11}^{Ge}	126 GPa
C_{12}^{Ge}	44 GPa

Substituting Eq. S2 in Eq. S3 results in:

$$\varepsilon_{\perp}^{GeSn} = (-2[xC_{12}^{Sn} + (1-x)C_{12}^{Ge}]/[xC_{11}^{Sn} + (1-x)C_{11}^{Ge}])\varepsilon_{||}^{GeSn} \qquad Eq.(S4)$$

The in-plane and out-of-plane strain of the GeSn film can be defined in terms of the unstrained lattice parameter by:

$$\varepsilon_{||,\perp}^{GeSn} = \left(a_{||,\perp}^{GeSn} - a_0^{GeSn}\right)/a_0^{GeSn} \qquad Eq.(S5)$$

Likewise, by substituting Eq. S1 in Eq. S5 results in:

$$\varepsilon_{||,\perp}^{GeSn} = \left[a_{||,\perp}^{GeSn} - xa_0^{Sn} - (1-x)a_0^{Ge}\right]/[xa_0^{Sn} + (1-x)a_0^{Ge}] \qquad Eq.(S6)$$

Therefore, by using Eq. S6, the relation described by Eq. S4 becomes:

$$\frac{[a_{\perp}^{GeSn} - xa_0^{Sn} - (1-x)a_0^{Ge}]}{[xa_0^{Sn} + (1-x)a_0^{Ge}]} = \left\{\frac{-2[xC_{12}^{Sn} + (1-x)C_{12}^{Ge}]}{[xC_{11}^{Sn} + (1-x)C_{11}^{Ge}]}\right\}\frac{\left[a_{||}^{GeSn} - xa_0^{Sn} - (1-x)a_0^{Ge}\right]}{[xa_0^{Sn} + (1-x)a_0^{Ge}]} \qquad Eq.(S7)$$

The terms of Eq. S7 can be reorganized to evidence its polynomial form:

$$(-a_0^{Sn}C_{11}^{Sn} + a_0^{Sn}C_{11}^{Ge} + C_{11}^{Sn}a_0^{Ge} - a_0^{Ge}C_{11}^{Ge} - 2a_0^{Sn}C_{12}^{Sn} + 2C_{12}^{Sn}a_0^{Ge} + 2a_0^{Sn}C_{12}^{Ge} - 2C_{12}^{Ge}a_0^{Ge})x^2 + \left(a_{\perp}^{GeSn}C_{11}^{Sn} - a_{\perp}^{GeSn}C_{11}^{Ge} - a_0^{Sn}C_{11}^{Ge} - C_{11}^{Sn}a_0^{Ge} + 2a_0^{Ge}C_{11}^{Ge} + 2a_{||}^{GeSn}C_{12}^{Sn} - 2C_{12}^{Sn}a_0^{Ge} - 2a_{||}^{GeSn}C_{12}^{Ge} - 2a_0^{Sn}C_{12}^{Ge} + 4C_{12}^{Ge}a_0^{Ge}\right)x + a_{\perp}^{GeSn}C_{11}^{Ge} - a_0^{Ge}C_{11}^{Ge} + 2a_{||}^{GeSn}C_{12}^{Ge} - 2C_{12}^{Ge}a_0^{Ge} = 0 \qquad Eq.(S8)$$

The roots of Eq. S8 can be determined through a change of variable, such as $x \equiv y + t, \forall y, t \in \mathbb{R}$, therefore Eq. S8 becomes:

$$
\begin{aligned}
&(-a_0^{Sn}C_{11}^{Sn} + a_0^{Sn}C_{11}^{Ge} + C_{11}^{Sn}a_0^{Ge} - a_0^{Ge}C_{11}^{Ge} - 2a_0^{Sn}C_{12}^{Sn} + 2C_{12}^{Sn}a_0^{Ge} + 2a_0^{Sn}C_{12}^{Ge} - 2C_{12}^{Ge}a_0^{Ge})y^2 \\
&\quad + (-a_0^{Sn}C_{11}^{Sn} + a_0^{Sn}C_{11}^{Ge} + C_{11}^{Sn}a_0^{Ge} - a_0^{Ge}C_{11}^{Ge} - 2a_0^{Sn}C_{12}^{Sn} + 2C_{12}^{Sn}a_0^{Ge} + 2a_0^{Sn}C_{12}^{Ge} \\
&\quad - 2C_{12}^{Ge}a_0^{Ge})2yt \\
&\quad + (-a_0^{Sn}C_{11}^{Sn} + a_0^{Sn}C_{11}^{Ge} + C_{11}^{Sn}a_0^{Ge} - a_0^{Ge}C_{11}^{Ge} - 2a_0^{Sn}C_{12}^{Sn} + 2C_{12}^{Sn}a_0^{Ge} + 2a_0^{Sn}C_{12}^{Ge} \\
&\quad - 2C_{12}^{Ge}a_0^{Ge})t^2 \\
&\quad + \big(a_\perp^{GeSn}C_{11}^{Sn} - a_\perp^{GeSn}C_{11}^{Ge} - a_0^{Sn}C_{11}^{Ge} - C_{11}^{Sn}a_0^{Ge} + 2a_0^{Ge}C_{11}^{Ge} + 2a_{||}^{GeSn}C_{12}^{Sn} \\
&\quad - 2C_{12}^{Sn}a_0^{Ge} - 2a_{||}^{GeSn}C_{12}^{Ge} - 2a_0^{Sn}C_{12}^{Ge} + 4C_{12}^{Ge}a_0^{Ge}\big)y \\
&\quad + \big(a_\perp^{GeSn}C_{11}^{Sn} - a_\perp^{GeSn}C_{11}^{Ge} - a_0^{Sn}C_{11}^{Ge} - C_{11}^{Sn}a_0^{Ge} + 2a_0^{Ge}C_{11}^{Ge} + 2a_{||}^{GeSn}C_{12}^{Sn} \\
&\quad - 2C_{12}^{Sn}a_0^{Ge} - 2a_{||}^{GeSn}C_{12}^{Ge} - 2a_0^{Sn}C_{12}^{Ge} + 4C_{12}^{Ge}a_0^{Ge}\big)t + a_\perp^{GeSn}C_{11}^{Ge} - a_0^{Ge}C_{11}^{Ge} \\
&\quad + 2a_{||}^{GeSn}C_{12}^{Ge} - 2C_{12}^{Ge}a_0^{Ge} = 0 \qquad Eq.(S9)
\end{aligned}
$$

The arbitrary dependence between y and t can be chosen so that Eq. S9 leads to the depressed polynomial form of Eq. S8. For that, the terms of Eq. S9 can be reorganized in terms of the order of y:

$$
\begin{aligned}
&(-a_0^{Sn}C_{11}^{Sn} + a_0^{Sn}C_{11}^{Ge} + C_{11}^{Sn}a_0^{Ge} - a_0^{Ge}C_{11}^{Ge} - 2a_0^{Sn}C_{12}^{Sn} + 2C_{12}^{Sn}a_0^{Ge} + 2a_0^{Sn}C_{12}^{Ge} - 2C_{12}^{Ge}a_0^{Ge})y^2 \\
&\quad + \big[2(-a_0^{Sn}C_{11}^{Sn} + a_0^{Sn}C_{11}^{Ge} + C_{11}^{Sn}a_0^{Ge} - a_0^{Ge}C_{11}^{Ge} - 2a_0^{Sn}C_{12}^{Sn} + 2C_{12}^{Sn}a_0^{Ge} \\
&\quad + 2a_0^{Sn}C_{12}^{Ge} - 2C_{12}^{Ge}a_0^{Ge})t + a_\perp^{GeSn}C_{11}^{Sn} - a_\perp^{GeSn}C_{11}^{Ge} - a_0^{Sn}C_{11}^{Ge} - C_{11}^{Sn}a_0^{Ge} \\
&\quad + 2a_0^{Ge}C_{11}^{Ge} + 2a_{||}^{GeSn}C_{12}^{Sn} - 2C_{12}^{Sn}a_0^{Ge} - 2a_{||}^{GeSn}C_{12}^{Ge} - 2a_0^{Sn}C_{12}^{Ge} + 4C_{12}^{Ge}a_0^{Ge}\big]y \\
&\quad + (-a_0^{Sn}C_{11}^{Sn} + a_0^{Sn}C_{11}^{Ge} + C_{11}^{Sn}a_0^{Ge} - a_0^{Ge}C_{11}^{Ge} - 2a_0^{Sn}C_{12}^{Sn} + 2C_{12}^{Sn}a_0^{Ge} + 2a_0^{Sn}C_{12}^{Ge} \\
&\quad - 2C_{12}^{Ge}a_0^{Ge})t^2 \\
&\quad + \big(a_\perp^{GeSn}C_{11}^{Sn} - a_\perp^{GeSn}C_{11}^{Ge} - a_0^{Sn}C_{11}^{Ge} - C_{11}^{Sn}a_0^{Ge} + 2a_0^{Ge}C_{11}^{Ge} + 2a_{||}^{GeSn}C_{12}^{Sn} \\
&\quad - 2C_{12}^{Sn}a_0^{Ge} - 2a_{||}^{GeSn}C_{12}^{Ge} - 2a_0^{Sn}C_{12}^{Ge} + 4C_{12}^{Ge}a_0^{Ge}\big)t + a_\perp^{GeSn}C_{11}^{Ge} - a_0^{Ge}C_{11}^{Ge} \\
&\quad + 2a_{||}^{GeSn}C_{12}^{Ge} - 2C_{12}^{Ge}a_0^{Ge} = 0 \qquad Eq.(S10)
\end{aligned}
$$

Therefore, the depressed polynomial form can be obtained by defining the arbitrary variable t so that:

$$
\begin{aligned}
&\big[2(-a_0^{Sn}C_{11}^{Sn} + a_0^{Sn}C_{11}^{Ge} + C_{11}^{Sn}a_0^{Ge} - a_0^{Ge}C_{11}^{Ge} - 2a_0^{Sn}C_{12}^{Sn} + 2C_{12}^{Sn}a_0^{Ge} + 2a_0^{Sn}C_{12}^{Ge} - 2C_{12}^{Ge}a_0^{Ge})t \\
&\quad + a_\perp^{GeSn}C_{11}^{Sn} - a_\perp^{GeSn}C_{11}^{Ge} - a_0^{Sn}C_{11}^{Ge} - C_{11}^{Sn}a_0^{Ge} + 2a_0^{Ge}C_{11}^{Ge} + 2a_{||}^{GeSn}C_{12}^{Sn} \\
&\quad - 2C_{12}^{Sn}a_0^{Ge} - 2a_{||}^{GeSn}C_{12}^{Ge} - 2a_0^{Sn}C_{12}^{Ge} + 4C_{12}^{Ge}a_0^{Ge}\big]y = 0 \qquad Eq.(S11)
\end{aligned}
$$

Therefore $\forall y \in \mathbb{R}$, the arbitrary variable t is given by:

$$t = -\big(a_{\perp}^{GeSn}C_{11}^{Sn} - a_{\perp}^{GeSn}C_{11}^{Ge} - a_0^{Sn}C_{11}^{Ge} - C_{11}^{Sn}a_0^{Ge} + 2a_0^{Ge}C_{11}^{Ge} + 2a_{||}^{GeSn}C_{12}^{Sn} - 2C_{12}^{Sn}a_0^{Ge} - 2a_{||}^{GeSn}C_{12}^{Ge} - 2a_0^{Sn}C_{12}^{Ge} + 4C_{12}^{Ge}a_0^{Ge}\big) /[2(-a_0^{Sn}C_{11}^{Sn} + a_0^{Sn}C_{11}^{Ge} + C_{11}^{Sn}a_0^{Ge} - a_0^{Ge}C_{11}^{Ge} - 2a_0^{Sn}C_{12}^{Sn} + 2C_{12}^{Sn}a_0^{Ge} + 2a_0^{Sn}C_{12}^{Ge} - 2C_{12}^{Ge}a_0^{Ge})] \qquad Eq.(S12)$$

Substituting Eq. S12 in Eq. S10 results in:

$$(-a_0^{Sn}C_{11}^{Sn} + a_0^{Sn}C_{11}^{Ge} + C_{11}^{Sn}a_0^{Ge} - a_0^{Ge}C_{11}^{Ge} - 2a_0^{Sn}C_{12}^{Sn} + 2C_{12}^{Sn}a_0^{Ge} + 2a_0^{Sn}C_{12}^{Ge} - 2C_{12}^{Ge}a_0^{Ge})y^2 + (-a_0^{Sn}C_{11}^{Sn} + a_0^{Sn}C_{11}^{Ge} + C_{11}^{Sn}a_0^{Ge} - a_0^{Ge}C_{11}^{Ge} - 2a_0^{Sn}C_{12}^{Sn} + 2C_{12}^{Sn}a_0^{Ge} + 2a_0^{Sn}C_{12}^{Ge} - 2C_{12}^{Ge}a_0^{Ge})\big\{-\big(a_{\perp}^{GeSn}C_{11}^{Sn} - a_{\perp}^{GeSn}C_{11}^{Ge} - a_0^{Sn}C_{11}^{Ge} - C_{11}^{Sn}a_0^{Ge} + 2a_0^{Ge}C_{11}^{Ge} + 2a_{||}^{GeSn}C_{12}^{Sn} - 2C_{12}^{Sn}a_0^{Ge} - 2a_{||}^{GeSn}C_{12}^{Ge} - 2a_0^{Sn}C_{12}^{Ge} + 4C_{12}^{Ge}a_0^{Ge}\big) /[2(-a_0^{Sn}C_{11}^{Sn} + a_0^{Sn}C_{11}^{Ge} + C_{11}^{Sn}a_0^{Ge} - a_0^{Ge}C_{11}^{Ge} - 2a_0^{Sn}C_{12}^{Sn} + 2C_{12}^{Sn}a_0^{Ge} + 2a_0^{Sn}C_{12}^{Ge} - 2C_{12}^{Ge}a_0^{Ge})]\big\}^2 - \frac{\begin{pmatrix} a_{\perp}^{GeSn}C_{11}^{Sn} - a_{\perp}^{GeSn}C_{11}^{Ge} - a_0^{Sn}C_{11}^{Ge} - C_{11}^{Sn}a_0^{Ge} + 2a_0^{Ge}C_{11}^{Ge} + 2a_{||}^{GeSn}C_{12}^{Sn} - 2C_{12}^{Sn}a_0^{Ge} - 2a_{||}^{GeSn}C_{12}^{Ge} \\ -2a_0^{Sn}C_{12}^{Ge} + 4C_{12}^{Ge}a_0^{Ge} \end{pmatrix}^2}{2(-a_0^{Sn}C_{11}^{Sn} + a_0^{Sn}C_{11}^{Ge} + C_{11}^{Sn}a_0^{Ge} - a_0^{Ge}C_{11}^{Ge} - 2a_0^{Sn}C_{12}^{Sn} + 2C_{12}^{Sn}a_0^{Ge} + 2a_0^{Sn}C_{12}^{Ge} - 2C_{12}^{Ge}a_0^{Ge})} + a_{\perp}^{GeSn}C_{11}^{Ge} - a_0^{Ge}C_{11}^{Ge} + 2a_{||}^{GeSn}C_{12}^{Ge} - 2C_{12}^{Ge}a_0^{Ge} = 0 \qquad Eq.(S13)$$

Therefore, by reorganizing Eq. S13, the variable y is:

$$y = \pm\sqrt{\left[\frac{\begin{pmatrix} a_{\perp}^{GeSn}C_{11}^{Sn} - a_{\perp}^{GeSn}C_{11}^{Ge} - a_0^{Sn}C_{11}^{Ge} - C_{11}^{Sn}a_0^{Ge} + 2a_0^{Ge}C_{11}^{Ge} + 2a_{||}^{GeSn}C_{12}^{Sn} - 2C_{12}^{Sn}a_0^{Ge} \\ -2a_{||}^{GeSn}C_{12}^{Ge} - 2a_0^{Sn}C_{12}^{Ge} + 4C_{12}^{Ge}a_0^{Ge} \end{pmatrix}^2}{4\begin{pmatrix} -a_0^{Sn}C_{11}^{Sn} + a_0^{Sn}C_{11}^{Ge} + C_{11}^{Sn}a_0^{Ge} - a_0^{Ge}C_{11}^{Ge} - 2a_0^{Sn}C_{12}^{Sn} + 2C_{12}^{Sn}a_0^{Ge} \\ +2a_0^{Sn}C_{12}^{Ge} - 2C_{12}^{Ge}a_0^{Ge} \end{pmatrix}^2}\right] - \left[\frac{a_{\perp}^{GeSn}C_{11}^{Ge} - a_0^{Ge}C_{11}^{Ge} + 2a_{||}^{GeSn}C_{12}^{Ge} - 2C_{12}^{Ge}a_0^{Ge}}{\begin{matrix} -a_0^{Sn}C_{11}^{Sn} + a_0^{Sn}C_{11}^{Ge} + C_{11}^{Sn}a_0^{Ge} - a_0^{Ge}C_{11}^{Ge} - 2a_0^{Sn}C_{12}^{Sn} + 2C_{12}^{Sn}a_0^{Ge} \\ +2a_0^{Sn}C_{12}^{Ge} - 2C_{12}^{Ge}a_0^{Ge} \end{matrix}}\right]} \qquad Eq.(S14)$$

Therefore, by using Eq. S12 and Eq. S14 in the original variable $x \equiv y + t$, the Sn content is given by:

$$x = -\left(a_{\perp}^{GeSn}C_{11}^{Sn} - a_{\perp}^{GeSn}C_{11}^{Ge} - a_{0}^{Sn}C_{11}^{Ge} - C_{11}^{Sn}a_{0}^{Ge} + 2a_{0}^{Ge}C_{11}^{Ge} + 2a_{||}^{GeSn}C_{12}^{Sn} - 2C_{12}^{Sn}a_{0}^{Ge} - 2a_{||}^{GeSn}C_{12}^{Ge} - 2a_{0}^{Sn}C_{12}^{Ge} + 4C_{12}^{Ge}a_{0}^{Ge}\right) /[2(-a_{0}^{Sn}C_{11}^{Sn} + a_{0}^{Sn}C_{11}^{Ge} + C_{11}^{Sn}a_{0}^{Ge} - a_{0}^{Ge}C_{11}^{Ge} - 2a_{0}^{Sn}C_{12}^{Sn} + 2C_{12}^{Sn}a_{0}^{Ge} + 2a_{0}^{Sn}C_{12}^{Ge} - 2C_{12}^{Ge}a_{0}^{Ge})]$$

$$\pm\sqrt{\left[\frac{\left(\begin{array}{c}a_{\perp}^{GeSn}C_{11}^{Sn} - a_{\perp}^{GeSn}C_{11}^{Ge} - a_{0}^{Sn}C_{11}^{Ge} - C_{11}^{Sn}a_{0}^{Ge} + 2a_{0}^{Ge}C_{11}^{Ge} + 2a_{||}^{GeSn}C_{12}^{Sn} - 2C_{12}^{Sn}a_{0}^{Ge}\\ -2a_{||}^{GeSn}C_{12}^{Ge} - 2a_{0}^{Sn}C_{12}^{Ge} + 4C_{12}^{Ge}a_{0}^{Ge}\end{array}\right)^2}{4\left(\begin{array}{c}-a_{0}^{Sn}C_{11}^{Sn} + a_{0}^{Sn}C_{11}^{Ge} + C_{11}^{Sn}a_{0}^{Ge} - a_{0}^{Ge}C_{11}^{Ge} - 2a_{0}^{Sn}C_{12}^{Sn} + 2C_{12}^{Sn}a_{0}^{Ge}\\ +2a_{0}^{Sn}C_{12}^{Ge} - 2C_{12}^{Ge}a_{0}^{Ge}\end{array}\right)^2}\right] - \left[\frac{a_{\perp}^{GeSn}C_{11}^{Ge} - a_{0}^{Ge}C_{11}^{Ge} + 2a_{||}^{GeSn}C_{12}^{Ge} - 2C_{12}^{Ge}a_{0}^{Ge}}{\begin{array}{c}-a_{0}^{Sn}C_{11}^{Sn} + a_{0}^{Sn}C_{11}^{Ge} + C_{11}^{Sn}a_{0}^{Ge} - a_{0}^{Ge}C_{11}^{Ge} - 2a_{0}^{Sn}C_{12}^{Sn} + 2C_{12}^{Sn}a_{0}^{Ge}\\ +2a_{0}^{Sn}C_{12}^{Ge} - 2C_{12}^{Ge}a_{0}^{Ge}\end{array}}\right]} \qquad Eq.(S15)$$

This relation can be simplified by defining the following parameters:

$$\alpha \equiv -\left(a_{\perp}^{GeSn}C_{11}^{Sn} - a_{\perp}^{GeSn}C_{11}^{Ge} - a_{0}^{Sn}C_{11}^{Ge} - C_{11}^{Sn}a_{0}^{Ge} + 2a_{0}^{Ge}C_{11}^{Ge} + 2a_{||}^{GeSn}C_{12}^{Sn} - 2C_{12}^{Sn}a_{0}^{Ge} - 2a_{||}^{GeSn}C_{12}^{Ge} - 2a_{0}^{Sn}C_{12}^{Ge} + 4C_{12}^{Ge}a_{0}^{Ge}\right) /[2(-a_{0}^{Sn}C_{11}^{Sn} + a_{0}^{Sn}C_{11}^{Ge} + C_{11}^{Sn}a_{0}^{Ge} - a_{0}^{Ge}C_{11}^{Ge} - 2a_{0}^{Sn}C_{12}^{Sn} + 2C_{12}^{Sn}a_{0}^{Ge} + 2a_{0}^{Sn}C_{12}^{Ge} - 2C_{12}^{Ge}a_{0}^{Ge})] \qquad Eq.(S16)$$

$$\beta \equiv \frac{a_{\perp}^{GeSn}C_{11}^{Ge} - a_{0}^{Ge}C_{11}^{Ge} + 2a_{||}^{GeSn}C_{12}^{Ge} - 2C_{12}^{Ge}a_{0}^{Ge}}{\begin{array}{c}-a_{0}^{Sn}C_{11}^{Sn} + a_{0}^{Sn}C_{11}^{Ge} + C_{11}^{Sn}a_{0}^{Ge} - a_{0}^{Ge}C_{11}^{Ge} - 2a_{0}^{Sn}C_{12}^{Sn} + 2C_{12}^{Sn}a_{0}^{Ge}\\ +2a_{0}^{Sn}C_{12}^{Ge} - 2C_{12}^{Ge}a_{0}^{Ge}\end{array}} \qquad Eq.(S17)$$

By using the values from Table S1, these parameters are approximately $\alpha = 2.0474a_{||}^{GeSn} + 3.96945a_{\perp}^{GeSn} - 2.16591$, and $\beta = 12.25654a_{||}^{GeSn} + 17.54914a_{\perp}^{GeSn} - 16.86405$.

Finally, the final expression for the Sn content ($0 \leq x \leq 1$) shown as Eq. 4 in the main text of the manuscript using RSM results of $a_{||,\perp}^{GeSn}$ is:

$$x = \alpha - \sqrt{\alpha^2 - \beta} \qquad Eq.(S18)$$

References

[1] M. Albani *et al.*, "Critical strain for Sn incorporation into spontaneously graded Ge/GeSn core/shell nanowires," *Nanoscale,* vol. 10, no. 15, pp. 7250-7256, 2018.
